# Beyond Traditional Use: The Scientific Evidence for the Role of *Astragali radix* in Organ Protection via Modulating Oxidative Stress, Cell Death, and Immune Responses

**DOI:** 10.3390/ph18101448

**Published:** 2025-09-26

**Authors:** Chuan Lin, Huiqiang Liu, Siyi Dong, Le Yang, Ling Kong, Yu Guan, Hui Sun, Guangli Yan, Ye Sun, Ying Han, Xijun Wang

**Affiliations:** 1 State Key Laboratory of Integration and Innovation of Classic Formula and Modern Chinese Medicine, National Chinmedomics Research Center, National TCM Key Laboratory of Serum Pharmacochemistry, Metabolomics Laboratory, Department of Pharmaceutical Analysis, Heilongjiang University of Chinese Medicine, Heping Road 24, Harbin 150040, China; 15983955737@163.com (C.L.); huiqanglliu@sina.com (H.L.); 15645408567@163.com (S.D.); 15244624557@163.com (L.K.); guanyuabout@126.com (Y.G.); gancaosuan@163.com (G.Y.); hanying314@sina.com (Y.H.); 2 State Key Laboratory of Dampness Syndrome, The Second Affiliated Hospital Guangzhou University of Chinese Medicine, Dade Road 111, Guangzhou 510180, China; leyang92gzy@sina.com (L.Y.); sunye0126@126.com (Y.S.)

**Keywords:** *Astragali radix*, oxidative stress, apoptosis, anti-inflammation, organ protection

## Abstract

Astragali radix (AR) is a traditional Chinese herbal medicine derived from the roots of *Astragalus membranaceus* and *A. mongholicus*. AR exhibits a wide range of pharmacological activities, such as cardioprotective, hypoglycemic, antitumor, antiviral, and multi-organ restorative effects. Nearly 400 bioactive compounds have been identified in AR by phytochemical investigations, with astragalus polysaccharides (APS), astragalosides (I–IV), formononetin (FMN), and calycosin (CA) established as principal bioactive constituents. These components exhibit multifunctional mechanisms encompassing antioxidative stress, apoptotic suppression, autophagy regulation, anti-inflammation, and immune regulation, thereby exerting significant protective effects on critical organ systems such as the cardiovascular, renal, neural, hepatic, gastrointestinal, and immune systems. This review synthesized research over the past three decades, elucidating the organ protective mechanisms of AR through phytochemical profiling. Key findings demonstrated that FMN-mediated Nrf2 pathway activation could attenuate reactive oxygen species (ROS) generation, while astragaloside IV (AS-IV) could suppress endoplasmic reticulum stress by inactivating the PERK/ATF6/CHOP axis to ameliorate apoptosis. Additionally, comprehensive safety assessment and pharmacokinetic analysis also substantiated favorable bioavailability and toxicological profiles. To sum up, these findings provide a comprehensive theoretical basis and offer innovative strategies for preventing and treating complex diseases associated with multi-organ dysfunction, thereby facilitating future clinical applications.

## 1. Introduction

*Astragali radix* (AR) is a perennial herb native to East Asia, including China, Japan, Mongolia, and the Korean Peninsula, which has been applied for over two millennia. The earliest medicinal use of AR was documented in Shennong’s Classic of Materia Medica, where it was classified as a superior-grade herb with a sweet taste, warm nature, and affinity for the lung and spleen meridians, with traditional functions such as tonifying Qi, elevating Yang, promoting diuresis, resolving edema, and nourishing blood [1]. According to the 2020 Chinese Pharmacopoeia, its medicinal material is the dried roots of *Astragalus membranaceus* (Fisch.) Bge. var. *mongholicus* (Bge.) Hsiao or *Astragalus membranaceus* (Fisch.) Bge. (Fabaceae). Currently, *A. membranaceus* var. *mongholicus* serves as the primary source of astragalus medicinal materials and is considered to possess marginally superior quality relative to other variants [2]. AR has been extensively utilized for managing cancer, diabetes, liver protection, and viral infections, as well as cardiovascular and cerebrovascular diseases [3,4,5,6]. In the practice of traditional Chinese medicine (TCM), AR is frequently applied in combination with other herbs to enhance the therapeutic efficacy through synergy while minimizing adverse effects. Representative synergistic formulations include Danshen–AR pairing for diabetic nephropathy [7], AR–Licorice decoction for microbiota-mediated liver injury amelioration [8], and the Shenbai formula (AR/Ginseng/Bupleurum) for treating postviral myocarditis sequelae and reducing side effects of conventional therapy [9].

In general, the maintenance of physiological homeostasis relies primarily on the fundamental functional units constituted by tissues and organs. The systemic equilibrium may be disrupted by structural compromise or functional impairment, potentially precipitating life-threatening pathologies. Recently, non-communicable diseases (NCDs) represent the predominant global health burden, accounting for 71% of worldwide mortality [10]. Representative types include malignancies, diabetes mellitus, chronic kidney disease, and cardiovascular disorders. These conditions can induce progressive organ damage through distinct pathophysiological mechanisms. For instance, chronic hyperglycemia in diabetes may accelerate end-stage renal disease, blindness [11,12], cerebrovascular accidents, and peripheral neuropathy [13,14], as it may drive microvascular compromise that produces negative impact on the vascular endothelium, renal tissue, retinal networks, and neural structures. Notably, phytochemicals demonstrate considerable organ protective potential. Astragaloside (AS) IV can attenuate diabetic nephropathy progression through coordinated modulation of renal AMPK/PI3K/AKT signaling, intestinal microenvironment restoration, glucose–lipid metabolism regulation, and mitochondrial protection [15]. Concurrently, the flavonoid calycosin (CA) can ameliorate glucocorticoid-induced femoral head osteonecrosis through dual osteometabolic regulation—suppressing inflammatory cascades via suppressing the TLR4/NF-κB pathway while promoting osteogenic differentiation and angiogenesis [16]. Collectively, via multi-targeted, multi-pathway synergistic mechanisms, natural plants or their extracts have unique advantages in protecting damaged organs and tissues, highlighting their values as promising candidates for disease prevention and treatment in the future.

To date, examination on AR have discovered approximately 400 secondary metabolites, including polysaccharides [astragalus polysaccharides (APS) I, II, and III], saponins (AS I–IV), flavonoids (baicalin, and quercetin), amino acids, and coumarins [17]. Pharmacologically, these bioactive constituents exert therapeutic effects by modulating key signaling cascades such as AMPK/PI3K/AKT, Nrf2/ARE, mTOR, and Wnt/β-catenin. Key mechanisms encompass antioxidant activity, anti-inflammatory responses, anti-apoptotic actions, autophagy inhibition, immune regulation, etc. So far, there is an absence of systematic reviews on AR-mediated multi-organ protective mechanisms. Therefore, we carried out a systematic review through literature retrieval across English and Chinese databases of PubMed, Web of Science, Google Scholar, and CNKI through June 2025 using “Astragalus”, “biological activities”, “apoptosis”, “autophagy”, “organ injury” and “organ protection”. With the integration of current in vitro and in vivo evidence, our comprehensive analysis intends to identify novel therapeutic strategies for AR-mediated tissue/organ protective mechanisms, so as to facilitate the clinical translation of AR-based therapeutic agents in organ injury repair.

## 2. AR Extracts and Chemical Properties

**Figure 1:**
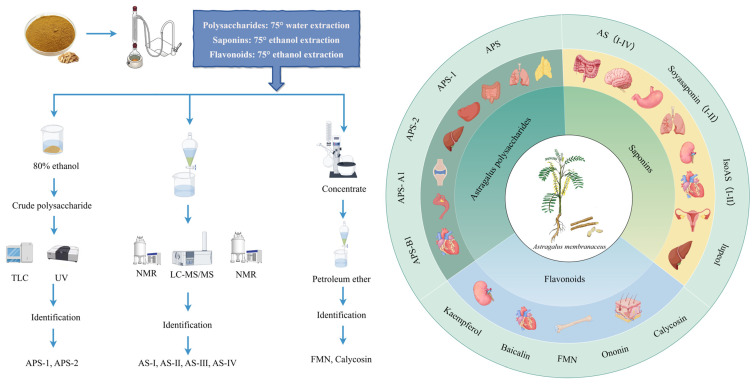
Process of extracting active ingredients from AR. This figure was drawn using figdraw (https://www.figdraw.com), export ID: AOORW8161e.

As mentioned above, we noted that there have been extensive phytochemical investigations over the past two decades, with the identification of over 400 bioactive constituents in AR, including polysaccharides, saponins, and flavonoids (Figure 1). These constituents—particularly astragalus polysaccharides (APS), AS, formononetin (FMN), and CA—have been recognized to be key therapeutic candidates given their protective effects on multiple organs through antioxidant, anti-inflammatory, antiapoptotic, and immunomodulatory activities. To gain a deeper mechanistic understanding of their organoprotective actions, these primary bioactive constituents were subjected to systematic characterization from the following perspectives of corresponding chemical structures, molecular weights, and extraction methodologies.

### 2.1. Polysaccharides

Acting as the principal bioactive macromolecules in AR, polysaccharides typically comprise high-molecular-weight polymers of glycosidically linked monosaccharides arranged in linear or branched configurations, with their structural architecture directly governing the pharmacological activity [18]. Heteropolysaccharides, glucans, and neutral/acidic polysaccharides constitute the predominant types among numerous polysaccharide classes isolated from AR. Among them, APS is generally isolated via water–alcohol extraction. Given their high polarity and aqueous solubility, crude APS at 10.97% efficiency can be yielded through hot water extraction at 75 °C followed by 80% ethanol precipitation [19]. According to compositional analysis via hot water extraction coupled with gas chromatography, the APS constituents were identified as l-rhamnose (l-Rha), d-xylose (d-Xyl), d-glucose (d-Glc), and d-galactose (d-Gal) in a 1:4:5:1.5 ratio. Structural characterization revealed a linear backbone in APS, consisting mainly of 1,3-linked b-D-Gal residues with the insertion of b-Glc, 1,6-linked a-Gal, 1,5-linked b-Xyl, 1,4-linked b-Gal, b-D-Gal, 1,2-linked a-Rha, and 1,2,4-linked a-Rha residues [20]. Chen et al. [21]. characterized two polysaccharides, designated APS-A1 and APS-B1, through infrared spectroscopy and nuclear magnetic resonance (NMR). Specifically, APS-A1, as a neutral polysaccharide, is composed of glucose, galactose, and arabinose, with a molar ratio of 52.3:1.0:1.3; while APS-B1 is a weakly acidic heteropolysaccharide composed of glucose, galactose, arabinose, xylose, rhamnose, and galacturonic acid, with a molar ratio of 75.2:17.3:19.4:1.0:1.1:1.3. Subsequently, this study provides an overview of other polysaccharides isolated and extracted from AR (Table 1).

### 2.2. Triterpenoid Saponins

With pharmacologically significant components of AR, saponins have demonstrated clinically relevant activities of cardiovascular protection, liver protection, antidiabetes, and anti-inflammation [31]. Depending on their chemical configurations, these triterpenoid saponins are categorized into cycloartane-type and oleanane-type structural classes. Among them, cycloartane-type saponins (80% of the total saponin content) prominently include AS I–IV and isoastragalosides I–II [32]. Currently, over 170 distinct saponins have been identified with the use of advanced analytical techniques, particularly liquid chromatography–mass spectrometry (LC–MS) and NMR. High-performance thin-layer chromatography–mass spectrometry has quantified AS I-IV content in root extracts at 0.2–0.5 mg/g [33,34]. Meanwhile, modern purification methodologies yield AS-IV at 96.95% purity [35] by employing high-speed countercurrent chromatography with ethyl acetate/n-butanol/water (4.2:0.8:5, *v*/*v*) two-phase systems, an ideal approach for AS-IV extraction. Table 2 summarizes the structural characteristics of the characterized Astragalus triterpenoid saponins.

### 2.3. Flavonoids

Flavonoids, categorized as flavonols, flavanones, anthocyanins, and isoflavones, constitute a prominent class of polyphenolic phytochemicals that are abundant in botanicals and human diets. These compounds exhibit significant anti-inflammatory, antiviral, antioxidant, and cardioprotective bioactivities [44]. Multiple flavonoid constituents in AR have been identified through phytochemical investigations. For instance, four flavonoid glycosides, i.e., 7-O-methylkaempferol 4′-O-β-D-galactopyranoside, hyperoside, isoquercitrin, and astragalin, were isolated in early studies employing chemical and spectroscopic methods [45]. Subsequently, two novel glycosides, i.e., kaempferol 7-methoxy-3-O-α-L-arabinosyl-(1→6)-β-D-galactopyranoside and kaempferol 3-O-α-L-rhamnopyranosyl-7-O-α-L-rhamnopyranosyl-(1→6)-β-D-galactopyranoside, were revealed by chromatographic study [45]. Moreover, Sheng et al. [46] isolated and identified flavonoid constituents from AR using methanol extraction, followed by UHPLC-MS/MS analysis. This facilitated the identification of 34 flavonoids, including well-known compounds (e.g., kaempferol, ononin, CA, and formonetin) that were confirmed in later research. Currently, water-to-methanol ratio optimization represents the predominant strategy for enhancing the extraction efficiency of flavonoids from AR [47]. Table 3 provides a comprehensive structural summary of Astragalus flavonoids characterized recently.

## 3. AR and Its Active Ingredients with Protective Effects on Multiple Organs and Tissues

AR primarily contains polysaccharides, saponins, and flavonoids according to modern phytochemical analyses. These constituents confer protective effects on multiple organ systems, including the intestinal tract, kidneys, joints, nervous system, cardiovascular system, liver, etc. These may be explained by diverse mechanisms, such as alleviating oxidative stress, modulating apoptosis pathways, regulating autophagy flux, attenuating immune responses, exerting anti-inflammatory effects, inhibiting ferroptosis and pyroptosis-mediated cell death, and displaying antifibrotic activity. This review continued to systematically summarize the evidence-based pharmacological characteristics and therapeutic applications of AR-derived compounds for specific organs, as shown in Table 2. Notably, a single active component (e.g., AS-IV) can target multiple organs (e.g., the heart, kidneys, and brain) via identical or distinct mechanisms, highlighting its multifunctionality. Conversely, there may be a formation of complex regulatory networks given that the protection of different organs frequently involves the coordinated regulation of multiple signaling pathways. Given the inherent complexity, it highlights the multicomponent, multitarget nature of AR, providing both a theoretical foundation and a significant research challenge for its clinical application in complex diseases.

### 3.1. Oxidative Stress

Oxidative stress refers to a pathological state arising from the imbalance between reactive oxygen species (ROS) and antioxidants that reduce ROS. This imbalance can trigger oxidative damage to biomolecules (e.g., lipids, proteins, and DNA) and cellular dysfunction [61]. Including both free radicals and non-free radicals, ROS are a class of oxygen-containing substances, such as superoxide anions (O_2_^−^), hydrogen peroxide (H_2_O_2_), hydroxyl radicals (·OH), ozone (O_3_), and singlet oxygen (^1^O_2_). Under normal physiological conditions, free radicals are generated through pathways such as the mitochondrial electron transport chain, NADPH oxidase (NOX), and xanthine oxidase in a controlled manner. Subsequently, these yielded free radicals may be promptly eliminated by antioxidant enzymes, such as superoxide dismutase (SOD) and glutathione peroxidase (GPx), thus maintaining a dynamic equilibrium. However, ROS accumulate exponentially and exceed the clearance capacity in the context of physical exposure to external stimuli (e.g., toxins, radiation, or inflammation), thereby leading to oxidative stress [62]. Through the Fenton reaction, excessive ROS can generate highly toxic ·OH that may induce lipid peroxidation, and destroy membrane fluidity and integrity. With further impact on mitochondrial membrane potential collapse, endoplasmic reticulum (ER) stress, and calcium overload, this damage can ultimately activate several apoptotic pathways (e.g., the caspase cascade) and necrotic apoptosis, resulting in parenchymal cell death and organ dysfunction.

**Figure 2:**
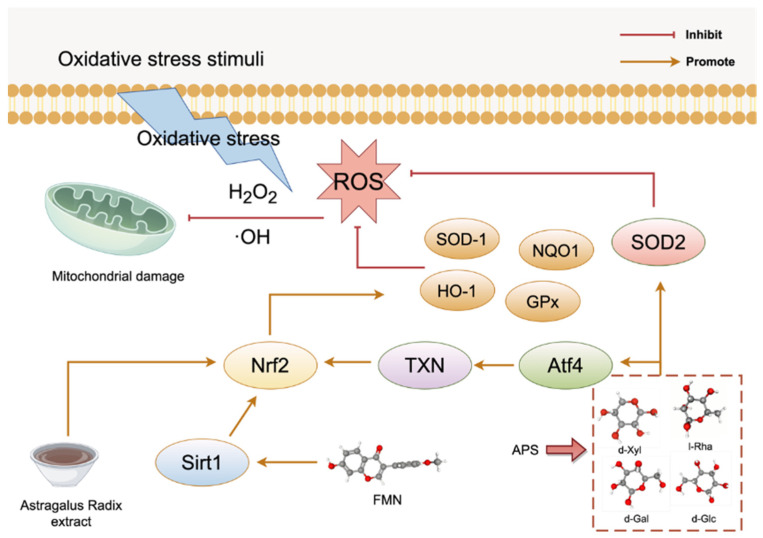
Molecular mechanism of the antioxidant action of AR. The APS constituents include l-rhamnose (l-Rha), d-xylose (d-Xyl), d-glucose (d-Glc), and d-galactose (d-Gal) in a 1:4:5:1.5 ratio. This figure was drawn using figdraw (https://www.figdraw.com), export ID: SROUT48444.

Numerous studies have extensively validated the antioxidant activity of AR, and have elucidated its involvement in antioxidant mechanisms. AR extracts exert antioxidant effects by reducing ROS release and activating antioxidant enzymes. For example, both lipopolysaccharide (LPS) from *E. coli* plus interferon-γ (IFN) inflammatory model and H_2_O_2_-induced oxidative stress model have revealed that AR extracts can effectively suppress the release of ROS. Furthermore, Nrf2 is a pivotal transcription factor antioxidant stress, plays a critical role in antioxidant responses [63]. AR extracts have been documented to exhibit significant antioxidant effects, as evidenced by the activation of Nrf2 and the substantial upregulation of two antioxidant enzymes of heme oxygenase-1 (HO-1) and NAD(P)H quinone oxidoreductase 1 (NQO1) [64]. The Nrf2/antioxidant response element (ARE) signaling pathway is a pivotal antioxidant signaling pathway within cells [65], which can be activated by silent information regulator 1 (Sirt 1). The flavonoid compound FMN present in AR has been reported to activate the Nrf2/ARE signaling pathway via upregulating Sirt1 expression, thereby enhancing the activity of antioxidant enzymes such as HO-1 and SOD-1 that are responsible for scavenging ROS. The result of this enhanced activity is a blockade of oxidative stress, which can improve the progression of diabetes and kidney disease [52]. In addition, AR-derived polysaccharides can reduce oxidative stress in osteoarthritis via a multifaceted process involving GCN2/TXN/NRF2. These proteins can activate the GCN2 kinase to sense oxidative stress and activate transcription factor (ATF) 4. ATF4 then upregulates the key antioxidant protein thioredoxin (TXN) to effectively suppress oxidative stress by scavenging ROS, thereby alleviating ROS-mediated DNA damage [66]. Figure 2 shows the specific factors and pathways involved in oxidative stress affected by the active components FMN and APS in AR.

Ji et al. [67] reported that baking AR at 260 °C for 30 min could elevate flavonoid concentration and provide neuroprotective effects through dual-pathway synergistic action. Roasting AR significantly increased the levels of catalase, HO-1, SOD-2, and glutathione peroxidase (GPX) by activating the Nrf2 pathway. It could further inhibit the release of ROS and offer neuroprotective effects by activating the brain-derived neurotrophic factor (BDNF) pathway and inhibiting neuronal apoptosis. It could also suppress the accumulation of beta-amyloid (Aβ)-induced neuronal oxidative damage and apoptosis.

Diabetic cardiomyopathy (DCM) refers to a myocardial metabolic dysfunction caused by the apoptosis of cardiac stem/progenitor cells (CSPCs) due to mitochondrial oxidative stress primarily induced by hyperglycemia-induced oxidative stress activation and elevated mitochondrial ROS levels [68]. Chen et al. [69] carried out an experiment with the use of Streptozotocin (STZ)-induced heterozygous (SOD-2^+/−^) knockout mice, where mitochondrial oxidative stress was stimulated with a 50% reduction in SOD-2 activity. The results revealed that the number of CSPCs decreased by 75–85% in mice with induced diabetes mellitus (DM) in the SOD-2^+/−^ group, and the DM-SOD-2^+/−^ group. Significantly intervention with APS led to 3.8- to 5.5-fold increase in the number of CSPCs. APS significantly reduced ROS production, as well as H_2_O_2_ and ·OH concentrations in CSPCs. Additionally, although the enzymatic and protein activities of SOD-2 in CSPCs were reduced in mice with DM, which were significantly increased after APS intervention. These findings indicate that APS specifically activates the SOD-2 pathway to inhibit mitochondrial ROS accumulation in diabetic hearts, highlighting a potential protective effect on CSPCs in diabetic patients. In addition, the production of ROS can be significantly influenced by glucose, lipids, succinate and other metabolic substrates [70]. For example, Jiang et al. found that AS-IV could alleviate myocardial ischemia–reperfusion injury by regulating succinate metabolism, inhibiting lysophospholipid accumulation, and activating the Nrf2/HO-1 antioxidant pathway [71]. In addition, CA was proven to reduce the levels of ROS and malondialdehyde (MDA) in myocardial tissue, enhance SOD activity, and mitigate oxidative stress-mediated myocardial cell necrosis in the isoproterenol-induced myocardial infarction model [72].

Therefore, AR enables the protection of the intestines [73], kidneys, joint cartilage, nervous system, heart [74,75], cardiovascular and skeletal muscles [76] by targeting oxidative stress-related signaling pathways and regulating the expression of related proteins, both upstream and downstream.

### 3.2. Antiapoptotic Effects

Apoptosis, similar to pyroptosis, necroptosis, and ferroptosis, is a genetically precisely regulated programmed cell death that is essential in maintaining tissue homeostasis, regulating normal development, and immune defense. In general, it is mainly mediated by the intrinsic mitochondrial pathway and the extrinsic death receptor pathway. Considering their differences in structural and functional characteristics, members of the Bcl-2 family can be classified into three categories, i.e., pro-survival proteins (e.g., Bcl-2, Bcl-XL, Bcl-w, Mcl-1, and A1); death effector proteins (e.g., Bax and Bak); and proapoptotic BH3-only proteins (e.g., Bid, Bim, Puma, Bad, and Noxa) [77]. In healthy cells, the death effectors Bax and Bak are bound by Bcl-2 proteins, whereas the proapoptotic BH3-only proteins are in an inactive state. Under the condition of cellular exposure to toxic stimuli, activated BH3-only proteins may release the bound Bax and Bak proteins, inducing mitochondrial outer membrane permeability and promoting the release of cytochrome c into the cytoplasm, in turn activating caspase-9 and effector caspases-3/7, ultimately resulting in cell disintegration [78]. Meanwhile, cytotoxic lymphocytes may produce death-inducing ligands in the extrinsic death receptor pathway. These ligands, involving FAS ligands, tumor necrosis factor (TNF), TNF-related apoptosis-inducing ligand (TRAIL), and TNF-α, can bind to specific death receptors on the target cell surface (FAS, DR4/DR5, and TNFR1, respectively), finally triggering the activation of caspase-8 directly [79].

Chk1 is a serine/threonine kinase that can modulate diverse cellular responses, relying primarily on its roles of controlling DNA damage, enforcing cell cycle checkpoints, and phosphorylating downstream substrates. Meanwhile, p53, a classical tumor suppressor, can activate its key downstream target p21, a cyclin-dependent kinase inhibitor. Both p53 and the p21 axis are central to coordinating the cellular response (e.g., cell cycle arrest and apoptosis) to DNA damage [80,81]. APS has been observed to attenuate paclitaxel-induced pro-apoptotic effects, which might be related to the reduced levels of phosphorylated H2A histone family member X (γH2AX; a DNA damage marker) and decreased PARP cleavage (an indicator of apoptosis execution). Furthermore, APS can upregulate the antiapoptotic proteins Bcl-XL and Mcl-1, and inhibits taxane-induced cell cycle arrest and apoptosis via downregulating the Chk1-p53-p21 signaling axis. This multifaceted mechanism underlies the ability of APS to selectively shield critical organs from paclitaxel toxicity. In addition, AS-IV (25–50 mg/kg) can suppress cell apoptosis through two pathways. On the one hand, it suppresses the extrinsic (death receptor) pathway by significantly downregulating the expression of Fas, Fas ligand (FasL), and Caspase-8 to inhibit the activation of downstream cleaved Caspase-3. Concurrently, it can inhibit the intrinsic (mitochondrial) pathway by remarkably heightening the Bcl-2/Bax ratio and reducing cytochrome c release from mitochondria [82]. In addition, AR extracts can retard oxidative stress-induced cell apoptosis by downregulating Bax expression, upregulating Bcl-2 expression, and restraining ROS production [83].

Simultaneously, AR also exhibits antitumor activity against diverse solid tumors. One of the major concerns in contemporary cancer research lies in overcoming chemoresistance and enhancing tumor sensitivity to therapeutic agents. In this context, dynamic and reversible posttranslational modification of O-GlcNAcylation has emerged as a critical regulator of cellular metabolism and signaling pathways, which facilitates rapid cellular adaptation to various stress stimuli [84]. O-GlcNAc transferase (OGT) and O-GlcNAcase (OGA) stand out as the two major enzymes regulating O-GlcNAcylation [85]. Specifically, hyper-O-GlcNAcylation, a posttranslational modification of proteins, occurs in the majority of malignant tumors, revealing positive correlation with tumorigenesis and progression [86]. Mechanistically, this modification engages in pathological crosstalk with ER stress across multiple cancers. ER stress propagates apoptosis through three distinct pathways: IRE1α, ATF6, and PERK, which independently induce apoptosis by activating apoptotic effectors, including caspase 12, c-Jun N-terminal kinase (JNK), ATF4, and the transcription factor C/EBP homologous protein (CHOP) [87]. For instance, APS impeded O-GlcNAc synthesis by decreasing OGT and augmenting OGA levels in doxorubicin (Dox)-treated Hep3B hepatocellular carcinoma (HCC) cell, leading to exacerbated Dox-induced ER stress, as evidenced by elevated CHOP expression. This study revealed that APS could promote apoptosis in Hep3B cells by downregulating Bcl-2 and upregulating Bax, Bim, and cleaved Caspase-3 through the CHOP-Bcl-2 pathway. Collectively, APS can increase the sensitivity of HCC cells to Dox [88]. Notably, Sun et al. [89] demonstrated that APS could specifically suppress the PERK-ATF6-CHOP ER stress axis to reduce cardiomyocyte apoptosis, thereby attenuating DCM. Moreover, peroxisome proliferator-activated receptor-γ coactivator 1 (PGC-1α) has been identified as a significant downstream target of Sirt1. FMN can modulate the homeostasis of mitochondrial dynamics (inhibiting fission/promoting fusion) through activating the Sirt1/PGC-1α pathway. This pathway works to restore mitochondrial function, attenuate ROS, normalize the Bcl-2/Bax ratio, and inhibit caspase-3-mediated renal tubular apoptosis [90].

In summary, AR exhibits potent antiapoptotic activity across multiple disease models, which is mediated mechanistically through coordinated regulation of both intrinsic (mitochondrial) and extrinsic (death receptor) apoptotic pathways. Altogether, APS shows promise in protecting the immune system, brain, liver, heart [91], kidneys, stomach [92], and joint cartilage by targeting key signaling pathways, such as OGT/OGA, PERK-ATF4-CHOP, and Chk1-p53-p21 [93].

### 3.3. Autophagy

Autophagy constitutes an evolutionarily conserved lysosomal degradation pathway that removes damaged organelles, misfolded proteins, and intracellular pathogens from the cytoplasm and recycles macromolecules into metabolic precursors to maintain eukaryotic cellular homeostasis [94]. This catabolic process begins with the formation of double-membrane autophagosomes that sequester targeted cell constituents and fuse with the lysosomess to form autolysosomes. Within these acidic compartments, lysosomal hydrolases can degrade encapsulated materials into reusable biomolecules (e.g., amino acids and nucleotides) to ensure energy homeostasis. Autophagy is precisely orchestrated by over 40 autophagy-related genes (ATG) through three core mechanistic stages. It can be subdivided into four stages: autophagy initiation, autophagosome formation, autophagolysosome fusion, and autolysosome degradation. To be specific, during initiation, Beclin-1 recruits VPS34 and PI3P to the autophagic precursor, thereby driving the formation of phagophores. ATG5 subsequently binds to ATG12 and recruits ATG16 to form the ATG5-ATG12-ATG16 complex, which can further polymerize on the surface of the phagophore membrane. During vesicle maturation, the developed complex synergistically promotes phagophore extension with LC3-II, which can further bind selectively to substrates awaiting degradation to guide the enclosure of materials by the autophagosome. At stages of terminal fusion and degradation, the V-SNARE subunits of the autophagosome membrane are anchored to the lysosome via VAMP proteins, whereas the STX17 subunit of the lysosome membrane forms the t-SNARE subunit with SNAP29. The assembly of these subunits to form a SNARE complex serves to mediate membrane fusion to facilitate the formation of autophagolysosomes. V-ATPase proton pumps then acidify these components, leading to the activation of cathepsin hydrolases that degrade contents into reusable metabolites, including amino acids and nucleotides, completing the autophagic flux that sustains cellular homeostasis [95].

The regulation of autophagy is orchestrated through integrated signaling–transcriptional networks with mTOR as the master inhibitory switch. Mechanistically, mTOR can competitively bind to ULK1 at the site of Ser^757^ to disrupt AMPK-ULK1 complex assembly, which may suppress the initiation of autophagy. Through negative feedback loops, the downstream ATG1 kinase complex can reciprocally regulate both autophagosome biogenesis and mTOR activity. The PI3K/Akt pathway, as an upstream regulator of mTOR, is inhibited during cellular stress or starvation to initiate autophagy [96]. Transcriptional control involves the MiT-TFE family (MITF/TFEB/TFE3/TFEC), which enables the activation of CLEAR-network genes for lysosomal biogenesis [97]; NF-κB, which possesses bidirectional regulatory function can be activated by ROS to induce autophagy under oxidative stress, and can also enhance autophagy through the mTOR pathway [98]; FOXO1/ATF4/HIF, which upregulates autophagy genes; and β-catenin, which suppresses core autophagic components via TCF4-mediated transcriptional repression. Collectively, all these findings may facilitate the establishment of a hierarchical control system that dynamically adjusts autophagy flux to metabolic and stress conditions [99].

**Figure 3:**
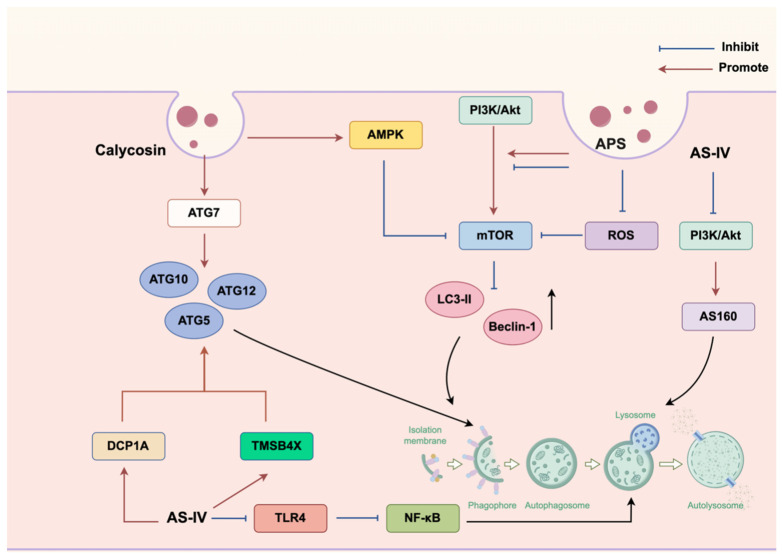
Molecular mechanisms of the anti-autophagy role exerted by AR. This figure was drawn using figdraw (https://www.figdraw.com), export ID: UPPUA29865.

Substantial evidence has documented that multiple bioactive constituents in ARs that modulate cellular autophagy pathways constitute a primary molecular mechanism underlying their organ-protective effects. Core components (e.g., APS, AS, and isoflavones) can regulate autophagy flux by targeting distinct signaling nodes. Specifically, APS can enhance autophagy via activating the PI3K/Akt/mTOR pathway, demonstrating therapeutic potential in Parkinson’s disease (PD) model [100]. Similarly, CA can promote autophagic clearance in osteoporosis [101]. Conversely, fermented AS preparations may inactivate PI3K/Akt/mTOR signaling, activating podocyte autophagy to mitigate hyperglycemia-induced damage [102]. Notably, this bidirectional regulatory capacity stems from the context-dependent functionality of mTOR. In other words, the overactivation of mTOR requires suppression to restore autophagy during metabolic stress (e.g., hyperglycemia), whereas moderate mTOR activation can initiate protective autophagy during cellular differentiation or proteostasis imbalance. Meanwhile, AS can suppress TLR4/NF-κB signaling and attenuate inflammatory responses in RAW264.7 macrophages to induce cytoprotective autophagy. Fluorescence microscopy confirmed AS-mediated autophagosome accumulation and autophagic vacuole formation, with impaired autophagy exacerbating the release of inflammatory cytokines [103]. In addition, by reducing ROS generation and downregulating LC3-II/Beclin-1 expression through ROS–mTOR pathway inhibition, APS can mitigate cadmium (Cd)-induced autophagic flux imbalance in chicken embryo fibroblasts (CEFs), thereby preserving cell viability [104]. AS160 is an effector protein of AKT that regulates the activity of Rab14, a member of the Ras superfamily and a core regulatory factor in eukaryotic intracellular transport. Rab14 has been reported to affect the activity of autophagy by modulating autophagosome–lysosome fusion [105]. Significantly, AS-IV can ameliorate chronic glomerulonephritis by enhancing autophagic flux by inactivating the PI3K/AKT/AS160 signaling axis [106]. Mechanistically, AS can induce a cascade of alterations, such as suppressing PI3K phosphorylation, reducing phosphorylated AKT (p-AKT) and AS160 (p-AS160) levels, and downregulating Rab14 expression. Eventually, it can facilitate the relief of Rab14-mediated inhibition of autophagosome maturation, promoting autophagolysosome formation and ultimately augmenting autophagic activity. CA can upregulate the expression of ATG7—a critical autophagy execution gene, which can contribute to the restoration of the autophagic homeostasis and mitigation of Dox-induced cardiotoxicity in delayed cardiac injury model [107]. Concurrently, CA can activate AMPK/mTOR signaling to initiate early autophagy while promoting the assembly of the STX17-SNAP29-VAMP8 SNARE complex, eventually restoring autophagosome–lysosome fusion and autophagic flux through dual-phase regulation. This mechanism can inhibit vascular smooth muscle cell osteogenic differentiation to attenuate vascular calcification [108]. This study plotted relevant autophagy regulatory pathways, as depicted in Figure 3. A pioneering study demonstrated that AS-IV could suppress the progression of cervical cancer by activating the Atg7/Atg12/LC3 autophagy axis through targeting DCP1A and TMSB4X. Quantitative proteomic profiling via iTRAQ-PRM technology identified 32 differentially expressed proteins (16 upregulated; and 16 downregulated), with mRNA decapping enzyme 1A (DCP1A) and thymosin β-4 (TMSB4X) emerging as significantly upregulated autophagy regulators. Mechanistically, DCP1A can accelerate autophagosome maturation through the WDFY3/Atg12 pathway, whereas TMSB4X may modulate autophagy initiation via the Akt/Atg5/Atg12 cascade [109].

Collectively, astragalus-mediated modulation of key autophagy pathways—including the PI3K/AKT/mTOR, TLR4/NF-κB, ROS-mTOR, PI3K/AKT/AS160, AMPK/mTOR, PINK1-Parkin, DCP1A/WDFY3/Atg12, and TMSB4X/Akt/Atg5/Atg12 pathways—can inhibit the therapeutic efficacy against multiple pathologies, such as PD, diabetes mellitus, osteoporosis, renal disorders, cardiovascular diseases, and cervical carcinoma.

### 3.4. Immunoregulatory Effects

The immune system is a fascinating, highly coordinated self-defense network comprising multicomponents of immune organs, immune cells, and immune-active substances that execute immune surveillance, defensive responses, and homeostatic regulation collectively [110]. The multifaceted roles of the immune system encompasses the defense against pathogen invasion and the fight against infection, beyond the identification and elimination of harmful substances within the body. Moreover, it is pivotal in slowing aging, maintaining cellular homeostasis, and combatting neoplasms [111].

AR exhibit immunomodulatory properties by modulating key immune organs such as the bone marrow [112], thymus [113], and spleen [112,113]. Meanwhile, it can enhance the activity of diverse immune cells, such as macrophages [113], natural killer (NK) cells [114], dendritic cells [112], T lymphocytes [115], lymphocytes [116], and oligodendrocytes [117]. AR can also promote the release of immune mediators, like interleukins (ILs; IL-2, IL-6, and IL-10), TNF-α, IFN-γ, and immunoglobulins (IgG and IgM) [113,118,119]. Furthermore, the programmed cell death protein 1 (PD-1)/programmed death-ligand 1 (PD-L1) axis represents a critical immune checkpoint in cancer, where PD-L1 binding to PD-1 suppresses T-cell proliferation, activation, and cytokine secretion. One of the key bioactive components of AR, APS, has significant immunomodulatory potential and is relevant to this pathway.

APS can activate both CD^4+^ and CD^8+^ T cells and reduce PD-L1 expression, as demonstrated in prostate cancer models [115]. Furthermore, APS can reactivate exhausted CD8+ T cells, inducing the secretion of pro-inflammatory factors, including IFN-γ, chemokine (C-X-C motif) ligand (CXCL) 2, chemokine (C–C motif) ligand 5 (CCL5), and CXCL12, thereby enhancing antitumor immunity. Concurrently, APS can stimulate macrophages to secrete cytokines such as TNF-α, IL-6, and CXCL2, which contribute to remodeling the tumor microenvironment toward an immunostimulatory state. Importantly, APS can benefit the reversal of T-cell exhaustion and the potentiation of existing immunotherapies by inhibiting PD-L1 expression and disrupting the PD-1/PD-L1 interaction [115].

Demyelination is a common pathological process underlying multiple neurological disorders, which is characterized by myelin sheath damage that may disrupt neurological function [120]. Currently, immunosuppressants, hormones, and other immunomodulatory drugs are the primary therapeutic options for the treatment of demyelinating diseases. However, these approaches often result in incomplete neural repair owing to their failure of promoting myelin regeneration directly [121]. Mechanistically, myelin restoration depends mainly on the differentiation of oligodendrocyte precursor cells (OPCs) into mature, myelinating oligodendrocytes (OLs) [122]. Concurrently, the Wnt/β-catenin signaling pathway has been documented to fundamentally orchestrate OPC development and myelination [123,124]. Yuan et al. [117] demonstrated that AS-II specifically bound to the Pro253 and Ser257 residues of p75NTR via hydrogen bonds. This binding would further stabilize the spatial conformation of p75NTR, enhancing its resistance to proteolytic degradation and thermal stability. However, noticeably, AS-II failed to promote myelin regeneration or improve motor function in p75NTR-knockout mice, confirming p75NTR dependence. Mechanistically, AS-II binding can promote OPC differentiation into myelinating OLs as it enables the potentiation of the GSK3β-mediated destabilization of β-catenin. By targeting this endogenous repair mechanism through indirect immune modulation, AS-II represents a novel broad immunosuppression-independent therapeutic strategy for myelin regeneration.

### 3.5. Anti-Inflammatory Effects

Inflammation is our body’s innate protective response to injury that functions significantly in the pathogenesis of diverse diseases. Chronic inflammation refers to a prolonged release of pro-inflammatory factors (e.g., TNF-α, IL-1β, and IL-6), chemokines, ROS, and proteases from activated immune cells (e.g., macrophages, neutrophils, and T cells) and tissue-resident cells. Due to excessive degradation or deposition (fibrosis), this persistent inflammatory response may culminate in tissue damage, cellular dysfunction, and dysregulation of the extracellular matrix, resulting in the disruption of the structural and functional integrity of the involved organs. Macrophages are central effectors of innate immunity, which can mediate crucial host defense functions (e.g., phagocytosis, cytokine secretion, and antigen presentation) against invading pathogens. Meanwhile, monocyte chemotactic protein-1 (MCP-1) is a chemokine that recruits monocytes, a type of immune cells involved in inflammatory responses, to the site of an inflammatory response [125]. Pioneering work by Chen et al. [21] isolated and purified two homogeneous polysaccharides (APS-A1 and APS-B1) from AR. The two polysaccharides have been demonstrated to significantly downregulate the mRNA levels and suppress the protein expression of key pro-inflammatory mediators, including TNF-α, IL-6, MCP-1, and IL-1β, in macrophages. This reduction effectively curbs the excessive secretion of these cytokines and inflammatory mediators. This anti-inflammatory activity involves central signaling pathways of NF-κB and MAPK, two representative regulators of inflammatory mediator expression [126,127]. Chen et al. [21] reported that APS-A1 and APS-B1 inactivated NF-κB and suppressed the phosphorylation of key MAPK kinases (JNK, ERK, and IKKα/β), thereby synergistically attenuating inflammation through the dual MAPK/NF-κB signaling axis. Furthermore, the NLRP3 inflammasome is a well-characterized critical pattern recognition receptor comprising NLRP3, ASC, and procaspase-1, which may contribute to diverse inflammatory pathologies upon dysregulated activation. With the resultant activation of autocatalytic caspase-1, this process can mediate proteolytic maturation and the release of pro-inflammatory cytokines IL-1β and IL-18 [128]. In addition to its role in intestinal inflammation, AS-IV can directly inhibit the activation of the NLRP3 inflammasome, which is evidenced by significantly attenuated protein expression of NLRP3, ASC, caspase-1, and NF-κB in the intestinal mucosa. Concurrently, AS-IV was observed to reduce the release of downstream IL-1β and IL-18, conferring significant protection against indomethacin-induced intestinal inflammation in rats [129]. As primary immune sentinels, macrophages constitute the first line of host defense, exhibiting functional polarization into two main phenotypes of the classically activated (pro-inflammatory M1) and alternatively activated (anti-inflammatory M2) macrophages [130]. Upon activation, M1 macrophages produce substantial levels of pro-inflammatory mediators, including IL-1β, IL-6, TNF-α, IL-12, ROS, and inducible nitric oxide synthase (iNOS). Conversely, M2 macrophages, induced by IL-4 and IL-10, can secrete anti-inflammatory factors such as arginase-1 (Arg-1). In vitro experiments revealed that APS inhibited the differentiation of macrophages into the pro-inflammatory M1 type, reduced the expression of M1 marker CD86, and decreased the release of ROS and the pro-inflammatory factors TNF-α, IL-6, and IL-12. Moreover, polysaccharides directly promoted the conversion of macrophages to the M2 type, upregulated the expression of M2 markers CD206 and Arg-1, and increased the secretion of anti-inflammatory factors IL-4 and IL-10. Critically, a relevant experiment based on gene silencing technique confirmed that the Nrf2/HO-1 pathway could mediate APS-induced macrophage phenotype regulation [131]. In a carrageenan-induced mouse paw edema model, astragalin also significantly alleviated acute inflammatory responses through dual mechanisms of scavenging ROS and inhibiting the NF-κB-mediated inflammatory pathway. Its anti-inflammatory efficacy is comparable to that of indomethacin (10 mg/kg), a common clinically used anti-inflammatory drug. As a naturally derived component, astragalin offers a greater safety advantage over synthetic anti-inflammatory drugs [132]. In addition to its anti-inflammatory effects, FMN can alleviate psoriasis-like inflammation. Mechanistically, FMN can downregulate the expression of key inflammatory factors TNF-α and IL-6 by impeding the IFN signaling pathway [129].

Collectively, APS, AS-IV, and FM, serving as the bioactive constituents of AR, can mitigate inflammatory responses through a multitargeted mechanism. This involves the strategic regulation of key inflammatory mediators (e.g., TNF-α, IL-6, IL-1β, IL-18, and MCP-1) and the concerted modulation of pivotal signaling pathways (e.g., NF-κB, MAPK, the NLRP3 inflammasome, Nrf2/HO-1, and IFN). On the basis of the proposed integrated molecular intervention, AR extracts and compounds confer significant protective effects across multiple organ systems, including the immune system (via macrophage polarization modulation), intestines, the vasculature, the skin, and lungs, demonstrating its broad therapeutic potential in inflammation-driven pathologies.

### 3.6. Other Functions

AR and its bioactive constituents exhibit significant antitumor activity, including anti-proliferative and pro-apoptotic effects, across a variety of cancer types. In particular, ovarian cancer (OC), one of the most prevalent and lethal gynecological malignancies, is characterized by high recurrence rates, metastasis, and a 5-year survival rate of only 25% [133]. MicroRNAs (miRNAs) are a family of small noncoding RNAs that serve as either oncogenes or tumor suppressors, which play crucial roles in the development of OC. Guo et al. reported [134] that through the modulation of the miR-27a/FBXW7 pathway, APS could inhibit proliferation and migration while promoting apoptosis in OC cell lines (OV-90/SKOV-3). Peng et al. revealed [135] that APS overcame PARP inhibitor resistance in OC stem cells (OCSCs) by targeting PINK1/Parkin-mediated mitophagy. Furthermore, AR-derived triterpenoid saponins (total Astragalus saponins, TAS) can also induce ferroptosis in gastric cancer, in addition to OC. Ferroptosis, an iron-dependent form of lethal lipid peroxidation-driven regulated cell death [136], can be activated by TASs through the SIRT3 pathway. TAS can modulate SIRT3 expression to trigger the remodeling of proferroptotic proteins, manifesting as the upregulation of ACSL4 alongside downregulation of SLC7A11 and GPX4. This imbalance can further elevate the levels of intracellular ROS, Fe^2+^, MDA, and lactate dehydrogenase, ultimately leading to ferroptotic cell death [137].

Conversely, AS-IV exhibits potent antiferroptotic activity in the model of cisplatin-induced hepatotoxicity. AS-IV can inhibit ferroptosis and mitigate liver injury through the activation of the PPARα/FSP1 axis. In vivo, AS-IV significantly reduced the levels of hepatic lipid peroxidation markers (4-HNE and MDA) while increasing the levels of the anti-ferroptotic factors GPX4 and FSP1 and suppressing the pro-ferroptotic enzyme arachidonate 15-lipoxygenase. In vitro studies have confirmed the efficacy of AS-IV in reversing cisplatin-induced cell death and lipid ROS accumulation through this mechanism. Mechanistic investigations revealed that AS-IV can directly bind to and activates peroxisome proliferator-activated receptor alpha (PPARα), upregulating its downstream effector ferroptosis suppressor protein 1 (FSP1). Crucially, pretreatment with either GW6471 (the PPARα antagonist) or iFSP1 (the FSP1 inhibitor) could completely abolish AS-IV-mediated cytoprotection, confirming the essential role played by the PPARα/FSP1 axis in exerting its antiferroptotic activity [138].

Pyroptosis represents another programmed cell death integral to innate immunity, which is an important contributor to the pathogenesis of inflammatory diseases [139]. In the context of asthma, AS-IV can exert certain therapeutic effects through promoting pyroptosis in airway smooth muscle cells (ASMCs). Mechanistically, AS-IV can disrupt its interaction with the receptor for advanced glycation end products (RAGE) based on its inhibitory role in the expression and cytoplasmic translocation of high-mobility group box 1 (HMGB1. By inactivating the NF-κB signaling pathway, this blockade can further trigger the assembly and activation of NLRP3 inflammasomes. Caspase-1 activation then mediates pyroptotic cell death in ASMCs, accompanied by the release of pro-inflammatory cytokines IL-1β and IL-18 [140]. Furthermore, AS-IV is effective against cholestatic liver fibrosis, a condition characterized by pathological bile acid (BA) accumulation, including tauro-conjugated BAs. The farnesoid X receptor (FXR), a BA-activated nuclear receptor, can regulate BA homeostasis via downstream effectors (e.g., SHP, Cyp7a1, NTCP, and BSEP). Zang et al. [141] reported that ARs could improve fibrosis, liver damage, and inflammation in cholestatic liver fibrosis by targeting the BA-FXR pathway. Specifically, as a multitarget antifibrotic agent, AS can significantly suppress the expression of the profibrotic markers α-smooth muscle actin and collagen type I alpha 1 while concurrently attenuating pathological collagen deposition in the liver tissue.

Collectively, AR-mediated cytoprotection orchestrates multiple programmed cell death pathways (e.g., ferroptosis, and pyroptosis), antifibrotic actions, and strategic modulation of critical signaling axes, including but not limited to the miR-27a/FBXW7, PINK1/Parkin-mediated mitophagy, SIRT3-dependent regulation, PPARα/FSP1, HMGB1/RAGE/NF-κB, and tauro-conjugated BA-FXR pathways.

### 3.7. Multipathway Synergistic Effects

The bioactive constituents of AR extracts confer multiorgan protection through synergistic modulation of various interconnected pathways. For instance, in alcoholic liver injury (ALI) with oxidative stress as a major contributor, APS and AS-IV can activate the KEAP1/NRF2 antioxidant pathway, enhancing the activity of endogenous antioxidant enzymes [e.g., SOD and glutathione peroxidase (GSH-Px)]. Concurrently, these compounds can directly scavenge excessive ROS and inhibit oxidative stress-induced mitochondrial apoptosis, as exemplified by suppressed activation of Bax [142]. Meanwhile, oxidative stress-derived ROS can further promote hepatic inflammation by stimulating the release of pro-inflammatory cytokines (IL-1β, IL-6, and TNF-α) [143]. Significantly, APS and AS work to mitigate this inflammatory response by downregulating the TLR4/MyD88/NF-κB signaling axis, significantly reducing TNF-α and IL-1β levels. This anti-inflammatory action alleviates tissue damage and indirectly suppresses caspase-mediated apoptosis triggered by inflammation. Critically, APS and AS facilitate the establishment of a synergistic antioxidant–anti-inflammatory network. Furthermore, the activation of NRF2 can inhibit NF-κB phosphorylation, whereas the suppression of NF-κB can reduce ROS generation, creating a self-reinforcing cytoprotective loop that amplifies the hepatoprotective effects [142]. Cycloastragenol (CAG) is a AS-derived bioactive aglycone that exhibits multimodal neuroprotective effects against PD through coordinated regulation of oxidative stress, autophagy, inflammation, and immune responses [144]. Mechanistically, CAG can attenuate oxidative damage through suppressing phagocytosis-induced ROS generation by inhibiting microglial Scribble and Scrib-p22^phox^ expression. Simultaneously, increased autophagic flux may promote the clearance of pathogenic α-synuclein (α-Syn), a hallmark protein in PD pathogenesis, and mitigate mitochondrial dysfunction. These mechanisms collectively inhibit NLRP3 inflammasome assembly via antioxidant/autophagy-mediated pathways, while increased autophagy directly degrades inflammasome components, ultimately reducing caspase-1-dependent gasdermin D cleavage and IL-1β/IL-18 release. Moreover, CAG can remodel the neuroimmune microenvironment by downregulating the expression of pro-inflammatory mediators (e.g., TNF-α, IL-6, and iNOS) and increasing the expression of neuroprotective markers (e.g., CD163), thereby driving microglial polarization toward a protective phenotype. Consequently, this phenotypic shift promotes the reduction of neuronal calcium overload, suppression of neuronal nitric oxide synthase activation, and inhibition of caspase-3-mediated apoptosis [144]. In addition, AR constituents confer renoprotection by modulating the PI3K/AKT/mTOR signaling axis, activating the NRF2/KEAP1/ARE antioxidant pathway, and suppressing TGF-β1/CTGF-driven fibrotic cascades, eventually leading to the preservation of the integrity of podocytes [145]. Table 4 lists the potential pharmacological effects of AR on different organs.

## 4. Safety Evaluation of AR Extract Components

AR, with a documented clinical history spanning nearly two millennia, is a cornerstone herb in TCM that possesses dual applications in medicinal and dietary contexts. As described in The Compendium of Materia Medica (Vol. 1, 2nd ed.), “AR, with edible stems, leaves, and flowers, is nontoxic.” With its inclusion in China’s “food-medicine homology” list, its modern regulatory recognition was affirmed in 2018, underscoring its safety profile as a natural product [147]. Pharmacological preparations—including aqueous decoctions and purified extracts (e.g., APS, AS)—demonstrate favorable safety within therapeutic dosages and a low incidence of adverse events [148]. Notably, a small-molecule compound isolated from AR, the telomerase activator TA-65, contains CAG as its primary bioactive aglycone. The neuroprotective efficacy of CAG, as evidenced by preclinical data, has been proven in murine models of neonatal hypoxic–ischemic brain damage (HIBD) [37]. In vivo studies demonstrated that TA-65 administration failed to increase telomere length in murine brains, coupled with no obvious effects on physiological parameters such as body weight, random blood glucose, hematological profiles, or hepatic/renal function markers. Moreover, TA-65 exhibited no significant modulatory effect on immune responses in HIBD models. In addition, chronic 24-week administration revealed no tumorigenic potential in major organs post-HIBD [149]. Furthermore, compound formulations, rather than isolated constituents, remain the major concern in current safety assessments of AR. Notably, a standardized AR-Panax notoginseng root extract blend indicated no genotoxicity in bacterial reverse mutation (Ames test) and murine bone marrow micronucleus assays. A 28-day repeated-dose (1200 mg/kg/day) oral toxicity study in rats revealed no mortality, target organ toxicity, or histopathological alterations, establishing a no-observed-adverse-effect level (NOAEL) of 1200 mg/kg/day [150]. Following single oral administration at 5000 mg/kg, the AR-containing polyherbal formulation HT042 (AR, *Eleutherococcus senticosus*, and *Perilla frutescens*) and its constituent botanicals exhibited no acute toxicity in Sprague–Dawley rats, with no observation of mortality or significant adverse effects. The LD_50_ exceeded 5000 mg/kg, satisfying the OECD Guideline 420 classification criteria for low-toxicity substances. In addition, in a 13-week subchronic toxicity assessment, no treatment-related mortality or clinically relevant toxicological alterations were induced through daily oral administration of HT042 at 4000 mg/kg/day (13-fold the clinical pediatric dose). Transient hematological variations (decreased red blood cell count and elevated reticulocytes/platelets) remained within the physiological range, whereas no correlative histopathological abnormalities were found in reversible organ weight increases (the spleen and kidneys). All these parameters were normalized during the 4-week recovery period, with the establishment of an NOAEL of 4000 mg/kg/day [151]. Altogether, these investigations confirm exceptional toxicological safety margins for AR-based formulations, maintaining biocompatibility at supratherapeutic exposures and supporting their clinical utility as functional ingredients.

## 5. Pharmacokinetic Study of AR

Pharmacokinetics (PK) offer a quantitative characterization of the absorption, distribution, metabolism, and excretion of xenobiotics within biological systems at the cellular, tissue, organ, and organismal levels. State-of-the-art analytical platforms, such as NMR, gas chromatography–mass spectrometry, and LC–MS, can quantify the temporal drug concentration profiles precisely. These methodologies can facilitate the establishment of critical parameters for clinical drug safety and efficacy given their roles in elucidating exposure–response relationships and mechanistic interactions in vivo. Critically, PK analysis provides a scientific basis for evaluating bioactive metabolite formation and potential toxicological implications [152].

PK profiling of AR bioactive constituents revealed the sustained-release characteristics underlying their therapeutic efficacy. For example, Xin Yu et al. [153] analyzed the plasma concentrations of six major components—calycosin-7-O-glucoside, ononin, methylnissolin-3-O-glucoside, AS–IV, and AS–II—in beagles following the oral administration of 4 g/kg aqueous extract using LC–MS. Complementary research by Jian Shi et al. [154] quantified eight analytes in rats after single-dose extract administration (4/16 g/kg). The panel included four parent compounds [calycosin-7-β-glucoside (CG), FMN, ononin, and AS-IV] and four phase II metabolites [calycosin-3′-glucuronide (C-3′-G), formononetin-7-O-glucuronide (F-7-G), calycosin-7-O-glucoside-3′-O-glucuronide (CG-3′-G), and formononetin-d-glucuronide (D-7-G)]. Consequently, rapid gastrointestinal absorption (T_max_ < 1 h) was observed, with the highest exposure (C_max_ > 1 μg/mL) observed in metabolite C-3′-G, followed by F-7-G. Collectively, saponins (notably AS-IV) and flavonoid metabolites exhibit prolonged systemic exposure through delayed absorption and extended elimination, revealing sustained pharmacological activities consistent with traditional dosing regimens. Another quantitative PK analysis revealed that AR aqueous extract significantly increased the bioavailability of six bioactive constituents: AS-I (LLOQ = 6.0 ng/mL), AS-II (9.6 ng/mL), AS-IV (11.8 ng/mL), calycosin-7-O-β-d glucoside (5.8 ng/mL), kaempferol (6.9 ng/mL), and FMN (9.4 ng/mL) compared with ultrafine powder (AP). Moreover, the administration of AP in the form of ultrafine powder outperformed the conventional intake of Huangqi water extract, which could increase the bioavailability of the compounds [155].

Combination therapy represents a cornerstone strategy for complex disease management. However, the presence of PK interactions [e.g., drug–drug interactions (DDIs) and herb–drug interactions [156] may potentiate therapeutic efficacy, induce cumulative toxicity, or compromise therapeutic outcomes. The cytochrome P450 (CYP) superfamily (notably CYP3A4, CYP2D6, and rodent-specific CYP2D1) remains central to these interactions, whose enzymatic activity governs xenobiotic metabolism and plasma concentration profiles. Thus, precise PK characterization elucidates metabolic interactions mediated by drug-metabolizing enzymes, revealing fundamental mechanisms of DDIs in PK [157]. Critically, AS-IV demonstrated inhibitory interactions with abemaciclib, a CDK4/6 inhibitor for treating breast cancer, upon coadministration [158]. For instance, through sampling from rats coadministered AS-IV and abemaciclib at various doses over 48 h, PK analysis of the plasma samples indicated that AS-IV dose-dependently potentiated systemic exposure to the CDK4/6 inhibitor. Specifically, AS-IV significantly increased the C_max_ and AUC_(0−t)_ of abemaciclib and eliminated t_1/2_. Table 5 summarizes the effects of AR and its extracts on PK parameters of various recent drugs. As a result, the increased concentration significantly reduced the activity of CYP3A4, indicating that AS-IV can enhance the therapeutic effect of abemaciclib by prolonging its in vivo half-life and reducing its clearance rate, thereby inhibiting its PK process. As demonstrated by the combination therapy addressing efficacy and safety concerns, AR injection can act synergistically with DOX, a broad-spectrum anthracycline antineoplastic agent limited by cardiotoxicity [159,160]. PK assessment via ultra-high-performance liquid chromatography with tandem mass spectrometry in male rats revealed that AR injection could obviously enhance the systemic DOX exposure, increasing the C_max_ from 2090.01 to 5262.77 ng/mL and the AUC from 1190.23 to 3777.27 μg/L × h [161]. Complementary in vitro microsomal studies also demonstrated CYP450 isoform-specific modulation, namely, inhibition of CYP1A2, CYP2C9, CYP2E1, and CYP3A4 while enhancing CYP2D1. This targeted CYP regulation can attenuate DOX metabolism, concurrently reducing toxicity (e.g., cardiotoxicity) and enhancing antitumor efficacy through improved PK profiles. Thus, these findings validate its role in mitigating DOX-associated adverse effects while enhancing therapeutic outcomes.

## 6. Conclusions and Future Perspectives

This review comprehensively summarizes the advances in in vivo and in vitro studies on AR, focusing on its major bioactive components identified over decades (polysaccharides, saponins, and flavonoids, e.g., APS, FMN, and CA) and their key pharmacological activities (e.g., organ/tissue protection, metabolic regulation, antioxidant effects, anti-inflammation, apoptosis modulation, and immune regulation). These components exert potentially effective therapeutic effects in disease management through the mediation of multiple signaling pathways such as Nrf2/ARE, PERK/ATF6/PI3K/AKT, and HMGB1/RAGE/NF-κB. This review also clarifies the in vivo PK properties of AR by analyzing the metabolism of its bioactive components (saponins, flavonoids), defining their PK characteristics and metabolic pathways, as well as revealing the modulatory role of AR in the metabolism of other drugs through the CYP450 enzyme system.

Current concern of research on AR involves its major compound classes, such as polysaccharides, flavonoids, and saponins. However, at present, there is still a notable lag in the analysis of individual active components, particularly with regard to the APS. To date, existing studies have identified several high-abundance monomeric polysaccharides (e.g., APS-1, APS-2, AH-1, and AH-2), yet with a poor understanding of their protective effects on organs and tissues. Further in-depth investigations are necessitated to unveil the mechanisms of action and corresponding target pathways of these individual components. Furthermore, the present study did not further discuss the active components in the stems and leaves of AR, despite relevant analyses on them in the rhizomes. It should be acknowledged that the stems and leaves also exhibit similar pharmacological effects, while extensive research has demonstrated the significant therapeutic potential of AR in various diseases. Yin et al. [164] pointed out that AS derived from these traditionally discarded aerial parts could improve cognitive function, reduce cerebral infarction volume, and attenuate neuronal apoptosis in a D-galactose- and AlCl_3_-induced mouse model of memory impairment. In addition, novel triterpenoid saponins isolated from AR leaves could also regulate mitophagy via the PINK1-Parkin pathway [165]. Crucially, accumulating evidence has supported the neuroprotective effects of AS-IV and triterpenoid saponins present in the roots and leaves of AR-producing plants, underscoring the therapeutic value of these plants in neuroprotection and highlighting their potential for rational utilization of medicinal plant resources.

Notably, our review underlines the multiorgan protective effects of the core bioactive constituents of APS, AS, and flavonoids by synergistically regulating oxidative stress, apoptosis, autophagy, immune, and inflammatory pathways. Specifically, this study systematically clarifies the molecular mechanisms of the “multi-component, multi-target, multi-pathway” synergistic organoprotective effect unique to AR, providing a valuable basis for developing novel AR-derived organ protectants (e.g., anti-fibrotic drugs, therapies against ischemia–reperfusion injury, and chemotherapy-induced toxicity mitigators) and designing clinical strategies integrating these bioactive components with existing therapeutic agents (e.g., Dox, abemaciclib) to enhance efficacy and reduce toxicity. Nevertheless, in view of the above discoveries and research gaps or limitations, further studies are required to define the structure–activity relationships of specific AR monomers, elucidate the precise mechanisms of multi-component synergy, and evaluate the clinical translational potential of lead compounds. Future research should prioritize key lead molecules (e.g., AS-IV, specific APS fractions, and flavonoids such as FMN) and couple this focus with rigorous clinical trials. Through the proposed targeted approach, it may be feasible to accelerate the development of safe, AR-derived organ protective drugs with specific actions, optimize therapeutic regimens for AR-containing formulations, and ultimately provide more effective therapeutic options for diabetic complications, cardiovascular/cerebrovascular disorders, organ fibrosis, and cancer adjuvant therapy.

## Figures and Tables

**Table 1:** Polysaccharides extracted from AR.

Source	Chemical Name	Composition	Quality Ratio	Molecular Weight (Da)	Classification		Ref.
Astragalus polysaccharides (APS)	APS	l-Rha:d-Xyl:d-Glc:d-Gal	1:4:5:1.5	3.01 × 10^5^	Heteropolysaccharides	Water-alcohol extraction	[20]
	APSID3	Ara:Rha:Gal:Glc	2:2:5:6	5.79 × 10^5^	—		[22]
	F-8	Rha:Rib:Fuc:Ara:Xyl:Man:Gal:Glc	2:2:1: 2:6:2: 3:100	2.2 × 10^4^	Neutral polysaccharide		[23]
	F-9	Fuc:Xyl:Glc	1:2:100	1.2 × 10^4^	—		[23]
	APS-1	Gal:Glc	1:49.76	3.84 × 10^4^	Heteropolysaccharides		[24]
	APS-2	Rha:Gal:Glc	1:2.99:16.26	5.2 × 10^3^	Heteropolysaccharides		[24]
	AH-1	Gly:Glc:Rha:Ara	1:0.04:0.02:0.01	—	Acidic polysaccharide		[25]
	AH-2	Glc:Ara	1:0.15	—	—		[25]
	APS-A1	Glc:Gal:Ara	52.3:1.0:1.3	2.62 × 10^6^	Neutral polysaccharide		[21]
	APS-B1	Glc:Gal:Arae:Man:Rha:GalA	75.2:17.3:19.4:1.0:1.1:1.3	4.95 × 10^6^	Acidic polysaccharide		[21]
	APS2-I	Man:Rha:GlcA:GalA:Glc:Gal:Xyl:Ara	2.3:4.8:1.7:14.0:5.8:11.7:2.8:12.6	1.96 × 10^6^	Dextran		[26]
	APS3-I	Rha:GlcA:Glc:Gal:Ara	0.8:2.3:0.8:2.3:4.1	3.91 × 10^6^	Dextran		[26]
	APS4	Rha:Ara:Xyl:Man:Gal	12.1:0.3:0.6:1.0:1.0:1.7	1.5 × 10^6^	—		[27]
	cAMPs-1A	Fuc:Ara:Gal:Glc:Xyl	0.01:0.06:0.20:1.00:0.06	1.23 × 10^4^	Water-soluble polysaccharides		[28]
	AAP-2A	Rha:Gal:Ara:Glc	1:2.13:3.22:6.18	2.252 × 10^3^	Neutral polysaccharide		[29]
	AERP1	Man:Rha:GalA:Glc:Gal:Ara	1.00:2.59:12.15:2.60:3.07:4.54	2.01 × 10^6^	Acidic polysaccharide		[30]
	AERP2	—	—	2.11 × 10^3^	Dextran		[30]

**Table 2:** Triterpenoid saponins extracted and isolated from AR.

Source	Chemical Names	Chemical Structure or Composition	Molecular Formula	Molecular Weight (Da)	Classification	Extraction Methods	Ref.
Astragalus saponins (AS)	AS-I	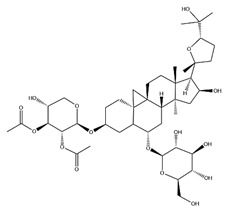	C_41_H_68_O_14_	869.04	Triterpenoid saponins	70% methanol extraction	[33,34]
	IsoAS-I	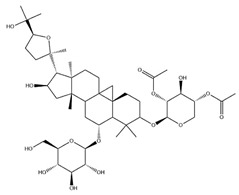	C_45_H_72_O_16_	869.02	Triterpenoid saponins	70% methanol extraction	[33,34,36]
	AS-II	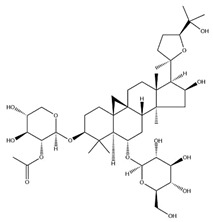	C_45_H_72_O_16_	827.13	Triterpenoid saponins	70% methanol extraction	[33,37]
	AS-III	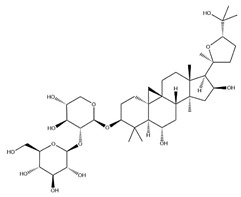	C_41_H_68_O_14_	784.98	Triterpenoid saponins	70% methanol extraction	[33,37]
	AS-IV	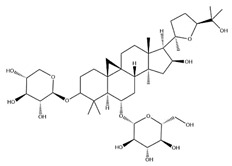	C_41_H_68_O_14_	784.98	Triterpenoid saponins	70% methanol extraction	[32,38,39]
	lupeol	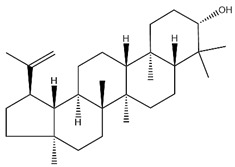	C_30_H_50_O	426.73	Triterpenoid saponins	Supercritical CO_2_ extraction	[40]
	Soyasaponin I	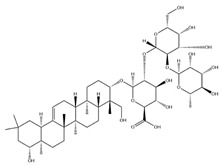	C_48_H_78_O_18_	943.26	Triterpenoid saponins	45% methanol solid phase extraction	[41]
	Soyasaponin II	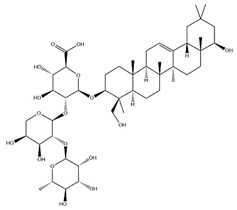	C_47_H_76_O_17_	913.11	Triterpenoid saponins	45% methanol solid phase extraction	[41]
	Agroastragaloside III	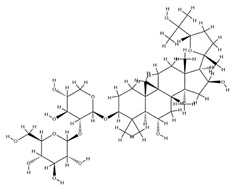	C_51_H_82_O_21_	1031.18	Triterpenoid saponins	Methanol extraction	[42]
	Agroastragaloside IV	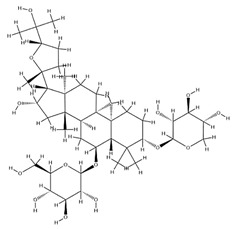	C_49_H_80_O_20_	989.14	Triterpenoid saponins	Methanol extraction	[42]
	Acetylastragaloside I	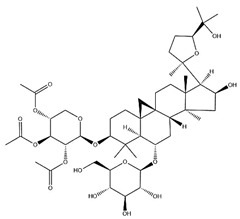	C_47_H_74_O_17_	911.10	Triterpenoid saponins	Methanol extraction	[43]
	IsoAS-II	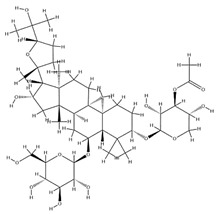	C_43_H_70_O_15_	827.02	Triterpenoid saponins	Methanol extraction	[43]
	AS-VII	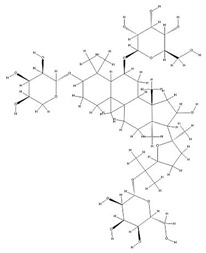	C_47_H_78_O_19_	947.1	Triterpenoid saponins	Methanol extraction	[43]

**Table 3:** Flavonoids extracted and isolated from AR.

Source	Chemical Names	Chemical Structure or Composition	Molecular Formula	Molecular Weight (Da)	Classification	Extraction Methods	Ref.
Flavonoids	Kaempferol	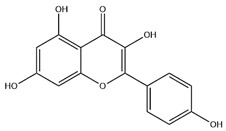	C_15_H_10_O_6_	286.24	flavonol	Ultrasound-assisted extraction (UAE)	[48]
	Baicalin	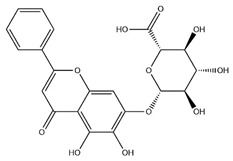	C_21_H_18_O_11_	446.36	Flavonoid glycosides	Microwave-assisted extraction (MAE)	[49]
	Isoliquiritigenin	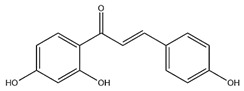	C_15_H_12_O_4_	256.28	Chalcone	UAE	[50]
	FMN	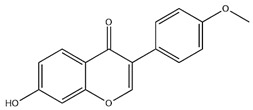	C_16_H_12_O_4_	268.26	isoflavone	Hexane-ethyl acetate-ethanol-water two-phase solvent extraction system	[51,52]
	Calycosin-7-O-β-d glucoside	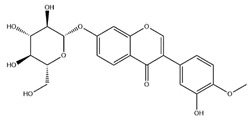	C_22_H_22_O_10_	446.40	isoflavone	70% ethanol extraction	[53,54]
	CA	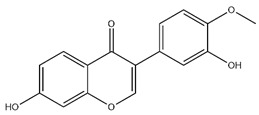	C_16_H_12_O_5_	284.28	isoflavone	70% ethanol extraction	[55]
	Ononin	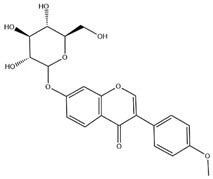	C_22_H_22_O_9_	430.40	isoflavone	Solid phase extraction (SPE)	[56]
	Quercetin	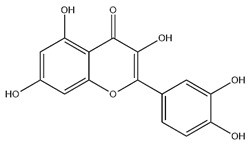	C_15_H_10_O_7_	302.24	flavonol	50% ethanol extraction	[57]
	Licochalcone B	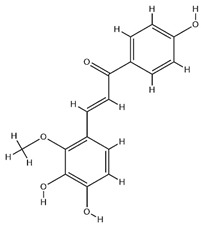	C_16_H_14_O_5_	286.28	Chalcone	Ethanol extraction	[58]
	Pendulone	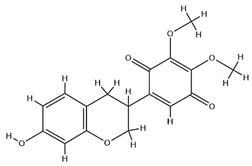	C_17_H_16_O_6_	316.31	Isoflavone	Ethanol extraction	[59,60]
	Astrasikokioside I	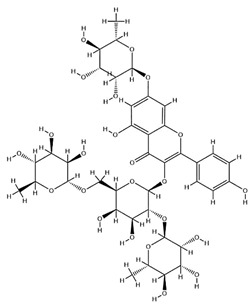	C_39_H_50_O_23_	886.80	Flavonoid glycosides	Ethanol extraction	[60]
	Isoquercitrin	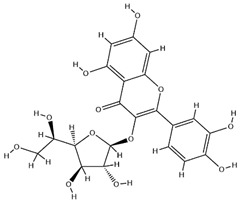	C_21_H_20_O_12_	464.38	Flavonoid glycosides	90% ethanol extraction	[45]
	Astragalin	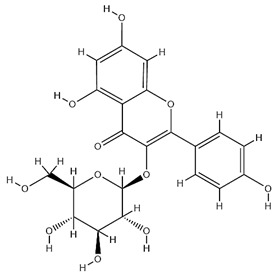	C_21_H_20_O_11_	448.38	Flavonoid glycosides	90% ethanol extraction	[45]

**Table 4:** List of potential pharmacological effects of AR on different organs. (Table Notes: ↑: increase; ↓: decrease).

Components	Models	Maximum Dose and Treatment Duration (In Vivo)	Organs	Effects	Mechanisms	Ref.
APS	Aβ25-35-induced HT22 mouse hippocampal neurons; Streptozotocin (STZ)-induced diabetic mouse model; and SOD-2^+/−^ gene knockout mouse model	2.0 g/kg; 10 weeks	Heart	Anti-oxidative stress; and apoptosis	↑SOD-2 enzyme activity; ↓ROS production, and ↓apoptosis in CSPCs.	[69]
APS	Chick transport stress (TS) model	100 μL/animal; 8 h (oral)	Heart	Anti-oxidative stress; and immunoregulation	↓mtDNA-PRRs pathway; ↑GSH, GPX, GST, SOD-2, and ↓MDA.	[74]
APS	Diabetic cardiomyopathy (DCM) rat model	1 g/kg; 16 weeks	Heart	Apoptosis	↓PERK and ATF6 pathways in ER stress.	[89]
CA	H9c2 cardiomyocytes subjected to thermal shock	—	Heart	Apoptosis	↓p-JNK pathway, Fas/FasL apoptosis pathway, and ↑PI3K/Akt pathway.	[91]
AS	Zebrafish	—	Heart	Autophagy	↑atg7, LC3-II, and ↓p62.	[107]
FMN	High-glucose-induced glomerular mesangial cells (GMCs); and db/db diabetic mice	25–50 mg/kg; 8 weeks	Kidneys	Anti-oxidative stress; anti-inflammation; and anti-fibrosis	↑Sirt1, and Nrf2/ARE pathway.	[146]
FMN	High-glucose-induced HK-2 human proximal tubule epithelial cell model; and STZ-induced diabetic nephropathy rat model	20 mg/kg; 8 weeks	Kidneys	Apoptosis	Regulation of Bcl-2/Bax balance, ↓caspase-3; as well as regulation of Sirt1/PGC-1α pathway and ↓ROS.	[90]
Fermentation of AR with P. cicadae	High-glucose-induced podocytes; and STZ-induced mouse diabetic nephropathy model	4.5 g/kg; 6 weeks	Kidneys	Autophagy	↓PI3K/AKT/mTOR pathway.	[102]
AS	LPS-induced human mesangial cell (HMC) model; and C-BSA-induced chronic glomerulonephritis (CGN) rat model	20 mg/kg; 6 weeks	Kidneys	Autophagy	↓PI3K/AKT/AS160, ↑C3-II, Beclin1, and ↓p62.	[106]
Astragalus injection/decoction	STZ-induced T1D, spontaneous GK rat model (T2D)	3–10 g/kg; 4–12 weeks	Kidneys	Anti-oxidative stress; and anti-fibrosis	↑NRF2/KEAP1 pathway, ↓TGF-β/Smad signaling, NF-κB activation, and regulation of the PI3K/AKT/mTOR pathway to protect podocytes.	[145]
APS	Hep3B liver cancer cells; and Hep3B cell-bearing nude mouse model	50 mg/kg; 28 days	Liver	Apoptosis	↓O-GlcNAc synthesis, ↑Dox-induced ER stress, ↓Bcl-2, and ↑activated CHOP and Caspase-3.	[88]
AS	AML-12 cell ferroptosis model; and cisplatin-induced mouse liver injury model	80 mg/kg; 9 days	Liver	Ferroptosis; anti-oxidative stress; and anti-inflammation	↓Ferroptosis; ↑PPARα/FSP1 signaling pathway, ↓key markers of ferroptosis, and regulation of lipid peroxidation.	[138]
ATS	Biliary duct ligation (BDL) rat cholestatic liver fibrosis model; and DDC diet-induced mouse liver fibrosis model	56 mg/kg; 4 weeks	Liver	Anti-fibrosis	↑FXR, SHP, BSEP, NTCP expression, ↓Cyp7a1, and reduce serum/liver taurine-conjugated bile acids (BAs).	[141]
APS, AS	Alcoholic liver disease (ALD) mouse model	APS: 600 mg/kg AS: 100 mg/kg; 4 weeks	Liver	Anti-oxidative stress; and anti-inflammation	↑KEAP1/NRF2 antioxidant pathway, and ↓TLR4/MyD88/NF-κB inflammatory pathway.	[142]
AS	Middle cerebral artery occlusion (MCAO) mouse model	40 mg/kg; 3 days	Spleen	Immunoregulation	Reduction of spleen atrophy and restoration of spleen NK/T/B-cell counts.	[116]
APS	4T1 breast cancer-bearing BALB/c mouse model	200 mg/kg; 14 days	Spleen, thymus	Immunoregulation; and apoptosis	Improvement of thymus index and spleen index, and enhancement of macrophage phagocytic ability.	[113]
AS	Cigarette smoke extract (CSE)-induced inflammatory model in RAW264.7 macrophages	—	Lungs	Autophagy; and anti-inflammation	↓TLR4/NF-Κb pathway, ↑LC3-II, ↑ATG5, ATG7, Beclin1; and ↓p62.	[103]
APS	Bleomycin (BLM)-induced model	100 mg/kg; 28 days	Lungs	Anti-inflammation	↓TLR4/NF-κB signaling pathway, TLR4/NF-κB pathway, TNF-α, IL-6, IL-1β, and TGF-β1.	[127]
Cycloastragenol (CAG)	Human gastric cancer SNU-1 and SNU-16 cells	—	Stomach	Apoptosis	↓STAT3 phosphorylation, JAK1/2, and Src kinase activity.	[92]
ATS	Gastric cancer cells (SGC-7901)	—	Stomach	Ferroptosis; and apoptosis	↑SIRT3 expression, ferroptosis, ↓SLC7A11/GPX4, and ↑ACSL4 protein.	[137]
AR water extract	IEC-6 intestinal epithelial cells; and mouse IBD model	—	Intestines	Anti-oxidative stress; and anti-inflammation	↓NF-κB pathway, and ↑Nrf2 pathway.	[64]
AS	DSS-induced mouse model of ulcerative colitis	100 mg/kg; 14 days	Intestines	Anti-oxidative stress; and immunoregulation	Regulation of Th17/Treg immune balance, ↓MDA, and ↑SOD/GSH-Px.	[73]
APS	LPS-induced RAW264.7 macrophages; and collagen-induced arthritis (CIA) rat model	200 mg/kg; 7 days	Intestines	Anti-inflammation	↓TLR4/NF-κB pathway, p-p65, p-IκB levels, MAPK pathway, IL-6, IL-1β, and TNF-α.	[126]
AS	Rat middle cerebral artery occlusion (MCAO) model	20 mg/kg; 7 days	Brain	Immunoregulation	Reduction of NK cell activation receptor NKG2D expression and IFN-γ production, and reversal of NK cell deficiency in the spleen and blood.	[114]
APS	BV2 microglia; and chronic fatigue syndrome (CFS) mouse model	800 mg/kg; 5 weeks	Gut; Brain	Anti-oxidative stress; and anti-inflammation	↑Nrf2 pathway; and ↓NF-κB pathway.	[75]
APS	6-OHDA-induced PC12 cells	—	Brain neurons	Autophagy	↑PI3K/AKT/mTOR pathway.	[100]
CAG	α-Synuclein (α-Syn)-induced Parkinson’s disease (PD) mouse model and primary microglia	125 mg/kg; 16 weeks	Central nervous system (Brain)	Anti-oxidative stress; and autophagy; Anti-inflammation; and immunoregulation	↓Scrib/NOX-ROS axis, inhibition of NLRP3, and ROS.	[144]
Processed AR water extract	Aβ25-35-induced HT22 mouse hippocampal neurons	—	Nervous system	Anti-oxidative stress; and apoptosis	↑Nrf2 pathway, and AKT/CREB/BDNF pathway.	[67]
APS	tBHP-induced mouse chondrocytes; and DMM surgery-induced osteoarthritis mouse model	—	Joint cartilage	Anti-oxidative stress	↑GCN2/ATF4/TXN axis.	[66]
APS	TBHP-induced chondrocyte apoptotic model; and DMM surgery-induced osteoarthritis mouse model	200 mg/kg; 6 weeks	Joint cartilage	Apoptosis	↓Mitochondrial apoptosis pathway; and ↓ROS.	[93]
AS	Sciatic nerve transection mouse model of muscle atrophy	20 mg/kg; 14 days	Skeletal muscle	Anti-oxidative stress; and anti-inflammation	↑SOD1/GPX1, ↓ROS/NOX2/4, ↓NLRP3/IL-1β/IL-6/TNF-α, and ↓LC3II/BNIP3	[76]
CA	Ovariectomized mice	50 mg/kg; 12 weeks	Bone tissue	Autophagy	↑PI3K/AKT/mTOR pathway.	[101]
AS	Experimental autoimmune encephalomyelitis model (EAE)	20 mg/kg; 10 days	Bone marrow	Immunoregulation	↓Maturation of splenic, bone marrow-derived DCs; ↓Th1/Th17 cell differentiation; ↓IL-6, and IL-12.	[112]
APS	Taxol-induced cytotoxicity in RAW 264.7 macrophages; and 4T1 tumor-bearing mouse model	40 mg/kg; 6 weeks	Immune system	Immunoregulation; and apoptosis	↓G2/M phase arrest, P-H2A.X, PARP, ↑Bcl-XL, and Mcl-1.	[82]
APS	LPS-induced RAW264.7 macrophages	—	Immune system	Anti-inflammation	↓NF-Κb, MAPK pathways; ↓ROS production (immune regulation); ↓NLRP3, iNOS, and COX-2 expression.	[21]
AS	Rat vascular smooth muscle cells	—	Vascular system	Autophagy	↑AMPK/mTOR pathway, and STX17-SNAP29-VAMP8.	[108]
APS	LPS/high-glucose-induced THP-1 macrophages and HUVECs coculture; and STZ-induced T2DM rat model	800 mg/kg; 8 weeks	Vascular endothelium	Anti-inflammation	↑macrophage M2 polarization, Nrf2/HO-1 pathway, ↓ROS, VCAM-1, MCP-1, and ↓Bax/Bcl-2.	[131]
APS	Ovarian cancer stem cells (OCSCs, 3AO/SKOV3 cells)	—	Ovaries	Autophagy	↓PINK1/Parkin pathway-mediated mitochondrial autophagy, and ↑TOMM20/COX IV, ↓PINK1 protein.	[135]
APS	OC cells (OV-90/SKOV3 cells)	—	Ovaries	Apoptosis	↓EMT; ↓miR-27a expression; ↑FBXW7 protein, and ↑E-cadherin.	[134]
AS	SiHa cell nude mouse xenograft model	50 mg/kg; 35 days	Cervix	Autophagy	Regulation of the DCP1A/WDFY3/Atg12 and TMSB4X/Akt/Atg5/Atg12 pathways.	[110]
AS	PDGF-BB-induced human airway smooth muscle cells (ASMCs); and OVA-induced asthma mouse model	100 mg/kg; 4 weeks	Respiratory tract	Pyroptosis	Alleviation of pulmonary inflammation; inhibition of HMGB1 cytoplasmic translocation, blockage of the HMGB1/RAG axis, inactivation of the NF-κB pathway, reduction of inflammatory factors, and ↑pyroptosis markers.	[140]
APS	Mouse macrophages (RAW 264.7), and mouse spleen-derived CD4^+^/CD8^+^ T cells	—	Prostate	Immunoregulation	Regulation of the PD-1/PD-L1 pathway, ↑T-cell secretion of IFN-γ, CXCL2, CCL5, TNF-α, IL-6, and CCL5.	[115]
APS	DSS-induced colitis mouse model	200 mg/kg; 14 days	Colon	Immunoregulation	↑Mitochondrial metabolism; ↑IgA^+^ MBCs, and ↓IgG^+^ MBCs.	[118]
AS	Indomethacin-induced SD rat enteritis model	80 mg/kg; 3 days	Small intestine	Anti-inflammation	↓NLRP3 inflammasome activation; ↓NF-κB4; ↓IL-1β, and IL-18.	[129]
FMN	HaCaT cells (psoriasis-like inflammation); and IMQ-induced psoriasis mouse model	—	Skin	Anti-inflammation	↓IFN-α/β/γ signaling pathway, CXCL9/10/11 and other chemokines, p-STAT1/3, IRF1 expression, TNF-α, IL-6, and IL-17 levels.	[119]
APS	Cadmium (CdCl_2_) induced chicken embryo fibroblast (CEF) damage model	—	Fibroblasts	Autophagy; and anti-oxidative stress	↓ROS, LC3-II, Beclin1, ↑SOD, and GSH-Px.	[104]
AS	Oligodendrocyte precursor cells; Cuprizone (CPZ)-induced mouse demyelination model, and experimental autoimmune encephalomyelitis (EAE) mouse model	50 mg/kg; 7 days	Myelin tissue	Immunoregulation	Targeting and binding to the p75NTR receptor, and ↓Wnt/β-catenin signaling pathway.	[117]

**Table 5:** Effects of AR and AR extracts on the PK parameters of drugs.

Medicines	Interacting Drugs	Animal Models	Routes of Administration and Dosages	PK Parameters of Ingredients							Ref.
				*C*_max_ (ng/mL)	AUC_(0−t)_ (ng/mL·h)	AUC_(0−inf)_ (ng/mL·h)	*t*_1/2_ (h)	*t*_max_ (h)	CL ((mL/h)/kg)	MRT_(0−t)_ (h)	
Astragali radix extract (ARE)	Dapagliflozin	Male SD rats	With ARE (300 mg/kg for seven days, intragastrically administered)	426.67 ± 58.92	4051.27 ± 952.61	4865.13 ± 1307.11	9.69 ± 3.47	1.50 ± 0.77	228.25 ± 56.10	7.64 ± 0.70	[162]
Astragalus Injection	DOX	Male SD rats	With Astragalus injection (4.25 mL/kg/day for 14 days, intraperitoneal injection)	5262.77 ± 111.15	3777.27 ± 130.55	7141.76 ± 177.96	0.14 ± 0.04	11.72 ± 1.22	1.09 ± 0.37	—	[161]
AS-IV	Abemaciclib	Male Sprague Dawley rats	With AS IV (50 mg/kg for 7d, orally administered	163,000 ± 147,850	36,380 ± 4230	—	38.24 ± 7.53	3.24 ± 1.15	0.49 ± 0.107	20.57 ± 0.92	[158]
			With AS IV (100 mg/kg for 7d, orally administered	1,698,170 ± 104,430	43,810 ± 1850	—	51.59 ± 17.08	2.65 ± 0.77	0.34 ± 0.059	20.87 ± 0.40	[158]
			With AS IV (150 mg/kg for 7d, orally administered	23,080,500 ± 55,290	66,140 ± 1170	—	66.17 ± 28.73	2.12 ± 0.84	0.19 ± 0.042	21.76 ± 0.32	[158]
Radix astragali	Pioglitazone	Male Wistar rats	With RA (28.35 g/kg for 7 days, orally administered)	2334.0 ± 87,369.92	9945.3 ± 871,556.29	—	2.787 ± 0.08	1.17 ± 0.42	36.55 ± 75.73	—	[162]
Huangqi injection (HI)	Gliquidone	Male Wistar rats	With HI (8 mL/kg for 5 min, intravenously)	22.46 ± 3.61	281.9 ± 12.4	—	2.46 ± 0.76	—	0.18 ± 0.04	—	[163]

## Data Availability

No new data were created or analyzed in this study. Data sharing is not applicable to this article.

## Abbreviations

AR	Astragali radix
APS	astragalus polysaccharides
FMN	formononetin
CA	Calycosin
ROS	reactive oxygen species
AS	astragalosides; astragalus saponins
AS-IV	astragaloside IV
IsoAS	isoastragalosides
SOD	superoxide dismutase
ATF4	transcription factor 4
TXN	thioredoxin
CSPCs	cardiac stem/progenitor cells
OGT	O-GlcNAc transferase
OGA	O-GlcNAcase
CHOP	C/EBP homologous protein
ER	endoplasmic reticulum
ATG	autophagy-related genes
DOX	doxorubicin
ASMCs	airway smooth muscle cells
CAG	Cycloastragenol
HIBD	hypoxic–ischemic brain damage
NOAEL	no-observed-adverse-effect level
PK	Pharmacokinetics
DDIs	drug–drug interactions
